# Mental Health Experiences Among Undergraduate Nursing Students in a New Zealand Tertiary Institution: A Time for Change

**DOI:** 10.1111/inm.13464

**Published:** 2024-10-31

**Authors:** Bernadette Solomon, Maia Topp, David J. A. Solomon, David Solomon

**Affiliations:** ^1^ Manukau Institute of Technology Auckland New Zealand; ^2^ Faculty of Health and Social Sciences University of Bedfordshire Luton UK; ^3^ Te Whatu Ora Health New Zealand Auckland New Zealand

**Keywords:** education, mental health, nurse training, psychosocial interventions, substance use

## Abstract

Nursing students in undergraduate programmes exhibit comparable, sometimes higher, levels of poor mental health and substance use compared to the general population; however, this area remains under‐researched in New Zealand. The study involved 172 nursing students enrolled in the Bachelor of Nursing programme at one tertiary institution in Auckland, New Zealand. Employing a mixed‐methodology approach, a 29‐question survey comprising both open and closed questions was administered to explore the students' experiences with mental health and substance use, as well as their access to support services. Quantitative data were analysed using SPSS version 29 descriptive statistics, while a general inductive approach guided the qualitative analysis. A significant proportion of participants (75%) reported experiencing emotional distress during their studies, with anxiety being the most prevalent (78.5%). A smaller percentage disclosed substance use (8.1%) including excessive alcohol use, cannabis use, nicotine use, vaping cannabis and some refusal to reveal substance use. Surprisingly, less than 1% (*n* = 0.6) utilised institutional support services. Three qualitative themes were identified including emotional distress and associated effects, emotional and psychological impacts on nursing students' academic journey and tertiary support systems. The findings highlight the urgent need to address the mental health and addiction challenges experienced by nursing students, given their potential adverse effects on academic success and overall well‐being. Urgent action is needed to integrate mental health training into the curriculum and provide faculty support. In this study, the underutilisation and inadequacy of institutional support services signal a need for institutional reforms to provide access and personalised mental health support to nursing students. Providing essential skills and support for student success contributes to the overall well‐being of the nursing workforce.

## 1 Introduction

In recent years, there has been a growing concern about the mental health and well‐being of undergraduate students, particularly within higher education institutions (Hemingway, Clifton, and Edward 2016). Campbell et al. (2022) highlighted a significant increase in depression and anxiety among college and university students, with stress levels and self‐harm rates on the rise. Various factors such as age, gender, ethnicity and sexual orientation have been identified as influencing mental health outcomes. Research indicates that mental health issues often manifest early in life, with a majority of young individuals experiencing symptoms by the age of 25 years (Kessler et al. 2007). Najafi et al. (2018) suggested a potential link between excessive internet use and negative mental health outcomes among nursing students. Socio‐economic factors, including rising tuition fees and financial pressures, also contribute to declining mental health, potentially leading to increased university dropout rates (Ladejo 2021).

Stigmatisation of mental health issues remains a pervasive issue within healthcare education, affecting students' attitudes and help‐seeking behaviours (Foster et al. 2019, 2021). Despite the availability of support services, many students hesitate to seek help due to concerns about stigma and fitness to practice as future nurses (Brown 2018). Loneliness and academic stress emerge as significant predictors of mental health issues among students, emphasising the need for comprehensive support systems within educational institutions (Corrigan, Druss, and Perlick 2014; Corrigan and Rao 2012; Foster et al. 2021; Lipson and Eisenberg 2017; McIntyre et al. 2018).

Addressing university students' mental health challenges requires a multifaceted approach, including awareness raising, stigma reduction and accessible support services. Creating a supportive and inclusive learning environment can enhance well‐being and academic success (Choi et al. 2016).

## 2 Methods

### 2.1 Study Design

Ethics approval was obtained from the Unitec Research Ethics Committee (XXX) Mātauranga Māori consultation was part of the mainstream research ethics process (Hudson et al. 2018). This study employed a mixed methods research approach, which facilitates the integration of both quantitative and qualitative research methods; thereby providing a holistic understanding of the research topic (Teddlie and Tashakkori 2009). The aims of the study were to understand:

1. The rates and associated experiences among student nurses regarding mental health issues and substance use.
2. What barriers exist for student nurses accessing their mental health support during their nurse training?
3. What supports student nurses' mental health during nursing training?

### 2.2 Setting

The study was conducted in a School of Nursing (SoN) based at a tertiary provider in Auckland, New Zealand. The SoN offers three, 3‐year degree programmes including the Bachelor of Nursing (BN), BN Pacific, BN Māori programmes.

### 2.3 Participants

Participants were BN students aged 18 years and above. An electronic poster with a survey link was distributed through learning platforms, facilitated by the SoN administrator. Lecturers provided project information to students. Implied consent was given when students read the research information and completed the anonymous questionnaire, which took approximately 20 min. A convenience sample of 172 student nurses, selected from a total population of 614 enrolled students across all 3 years of the BN programme, participated anonymously.

### 2.4 Data Collection

The questionnaire, a novel instrument, resulted from a synthesis of a range of sources, including the research team's expertise, extensive literature review and consultations with mental health nursing experts. This questionnaire was specifically developed for a single study to explore undergraduate nursing students' mental health and substance use at a single tertiary location in Auckland, New Zealand. The questionnaire aimed to identify key content domains including experiences with mental health issues, the impact of these on their daily lives and academic study, substance use and support resources they used. Each domain was theoretically defined in the questionnaire. Questionnaire items were created to align with these domains, drawing on insights from relevant literature and related instruments. The items were carefully reviewed to eliminate any overlap or duplication, resulting in a refined set of items designed to explore the rates and associated experiences of mental health and substance use among student nurses within this tertiary institution.

In the next phase, face validity was assessed by four content experts, including academics with experience in instrument development and topic experts. Through email correspondence and in‐person meetings, this panel thoroughly examined the study's objectives, reviewed the survey instrument and evaluated the clarity of the questions and instructions provided to respondents. These experts provided feedback regarding the instrument, assessing whether the instrument appeared to measure what it was designed to measure (i.e. its face validity; Gaber 2010).

Additionally, two separate independent reviewers also provided feedback on the face validity of the instrument as part of the ethics approval process. Based on their recommendations, specific items were modified to enhance the instrument's effectiveness. Both reviewers agreed on these changes, ensuring that the final questionnaire had strong face validity and was well‐suited for its intended purpose.

The questionnaire design encompassed three main categories of questions; target, administrative and clarification (Al‐Ababneh 2020; Cooper and Schindler 2014). It consisted of 29 questions covering demographic characteristics (e.g. age, gender, year in programme, relationship status, living situation, ethnicity and parental status) and mental health topics that explored students' mental health including anxiety, emotional distress, physical well‐being, substance use, self‐harm, suicidal thoughts, support systems and suggestions for institutional support. Participants responded by selecting ‘yes’, ‘no’ or ‘prefer not to say’; with open‐ended questions providing participants the opportunity to elaborate on their experiences. A 4‐point Likert scale measured overall mental and physical health. Data were securely stored in a dual password‐protected cloud system accessible only to the research team.

### 2.5 Data Analysis

Quantitative data analysis was conducted using SPSS version 29 statistical analysis software. Descriptive analysis was performed on demographic variables such as age, gender, and programme year. The prevalence of mental health issues among student nurse respondents including anxiety, emotional distress, physical well‐being, substance use, self‐harm, and support systems was examined. Qualitative short‐answer responses within these categories, as well as suggestions for institutional support, were analysed using Thomas' (2006) general inductive approach thematic analysis method. It involved drawing out relevant quotes and extracts from the survey and identifying suitable quotes of thematic understanding, which helped to identify commonalities and variations within and across participants.

## 3 Findings

### 3.1 Quantitative Results

Out of 614 students enrolled in the BN programme, 172 (29%) nurses completed the questionnaire. Among these respondents, were 42 Year 1 students, 71 Year 2 students and 59 Year 3 students. The majority of participants were female (*n* = 152, 88.4%), with 11% (*n* = 19) identifying as male and 0.6% (*n* = 1) undisclosed. The largest age group was 18–29 years (*n* = 111, 64.5%), followed by the 30–39 age bracket (*n* = 38, 22.1%) and the 40+ age group (*n* = 23, 13.4%). European was the most common ethnicity (*n* = 61, 35.5%), followed by Asian (*n* = 35, 20.3%), South Asian (*n* = 31, 18%), Pacific (*n* = 27, 15.7%) and other (*n* = 11, 6.4%). Regarding relationship status, 34.9% (*n* = 60) reported being single, 33.1% (*n* = 57) reported being in a relationship, 26.2% (*n* = 45) were married, 2.3% (*n* = 4) divorced and 3.5% (*n* = 6) preferred not to say. Most of the participants either lived with their parents (*n* = 74, 43.3%) or in rented accommodation (*n* = 65, 38%), 17% (*n* = 29) owning their own home and 1.2% (*n* = 2) living with other relatives. Most participants did not have children (*n* = 119, 70%; see Table 1).

**TABLE 1:** Survey demographics, frequencies and free‐text questions.

Variables	Nursing students demographics	Frequencies (*N*)	Free‐text survey questions
Total participants	Students	172	Overall, how do you rate your physical health? Overall, how would you rate your mental health? During the past year, how have you experienced any issues with your work or daily life due to any emotional distress (such as feeling depressed, sad or anxious) If you answered yes, please state which issue During the past year, have you experienced any mental health or addiction/ mental health challenges? If you answered yes, please state which issue During the past year, how have you experienced or struggled with substance use? If yes, please share what type of use and how it affects you Are you currently taking any medication to support your mental health? Do you experience (symptoms) of schizophrenia? If yes, please share these symptoms Do you experience Bipolar affective Disorder? If yes. Please share these symptoms. Do you experience any one of the following symptoms? Anxiety, for example, constant worry, unable to relax, panic sensations Have you ever felt like hurting yourself? Do you experience thoughts/ feelings of suicide or have self‐harmed? What helps your mental health? Do you know what services are available to support you with your mental health at XXX Please say below. Does your emotional/ psychological challenges affect your study? If you answered yes, please state below What other mental health support do you think would be beneficial whilst you are studying nursing at XXX Do you want to share any other information?
Gender	Male	152	
	Female	19	
Prefer not to say	Male/Female	1	
Age	18–29 years	111	
	30–39 years	38	
	40+	23	
Ethnicity	European	61	
	South Asian (Indian, Fijian Indian)	31	
	Māori	6	
	Pacific (Samoan, Tongan, Cook Island Māori, Fijian, Niuean)	27	
	Asian (Filipino, Cambodian, Vietnamese, Chinese)	35	
	Other (South African, African, Kurdish, European/Tongan, Peruvian)	11	
	Prefer not to say	1	
Year of nursing study	Year 1	42	
	Year 2	71	
	Year 3	59	
Relationship status	Single	60	
	Married	45	
	In a relationship	57	
	Divorced	3	
	Prefer not to say	7	
Accommodation	Home owner	29	
	Living with parents	74	
	Rent/boarding	65	
	Living with other relatives	2	
	Alternative accommodation	1	
	Dependent children		
	Yes	51	
	No	119	

### 3.2 Psychological Health

**GRAPH 1:**
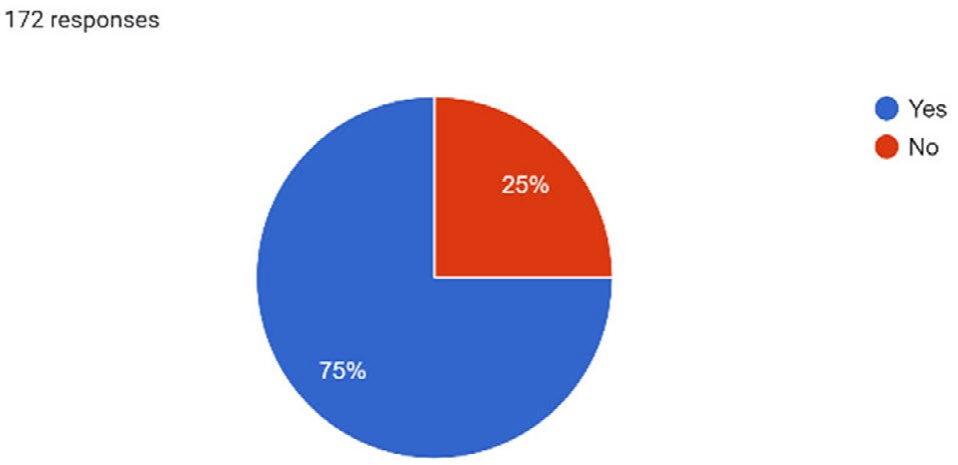
Emotional Distress.

**GRAPH 2:**
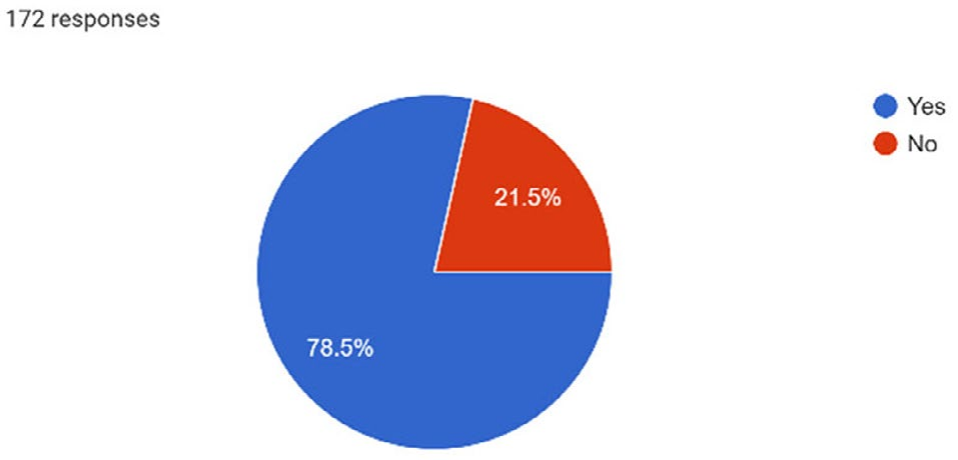
Anxiety symptoms.

**GRAPH 3:**
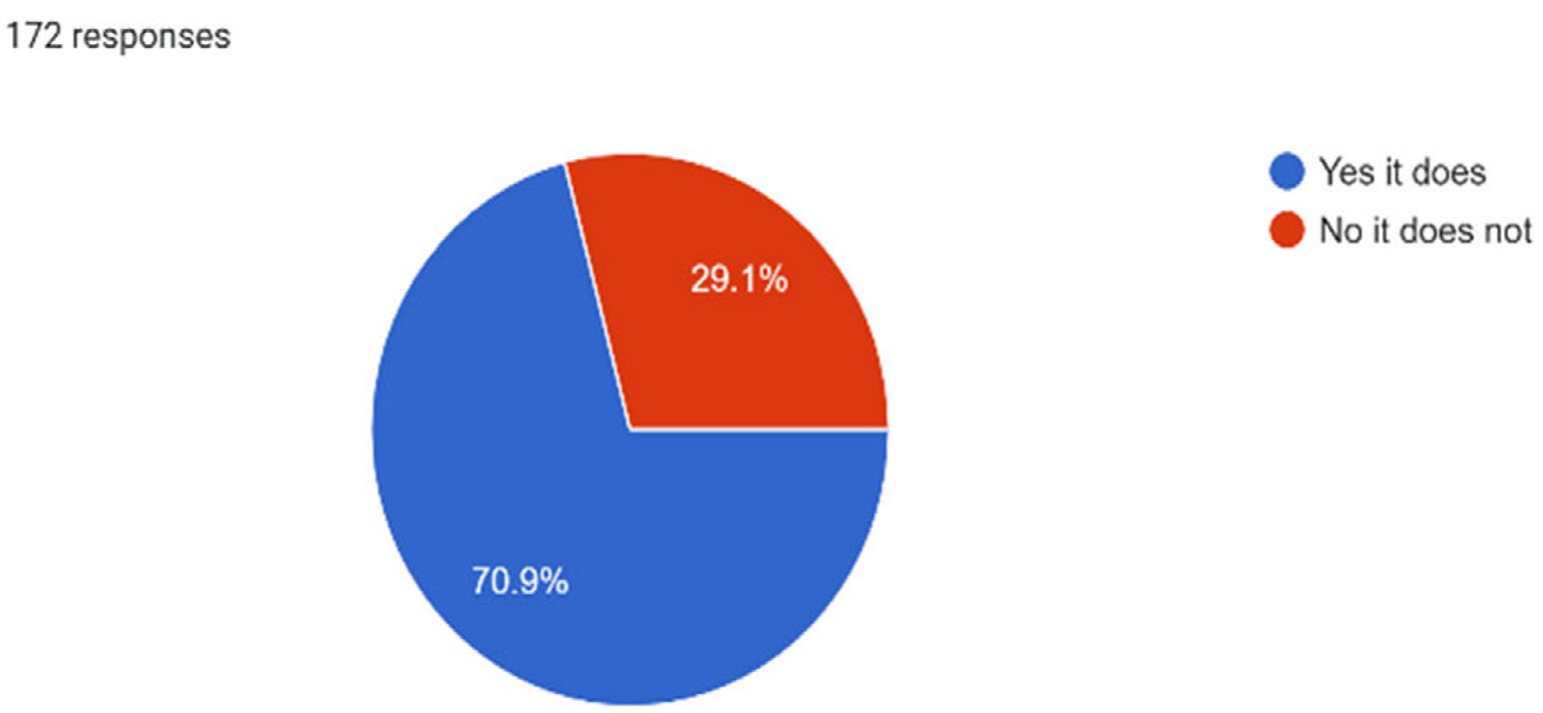
Psychological challenges.

Just under half (45.4%) of the study population reported their mental health as good to excellent, while the remaining half (54.6%) rated it as somewhat good or poor. Among those, 75% acknowledged experiencing emotional distress such as feeling depressed, sad, or anxious within the past 12 months (Graph 1). Regarding anxiety symptoms, 78.5% of participants affirmed experiencing constant worry, inability to relax or panic sensations (Graph 2), with 70.9% reporting that emotional distress had an impact on their academic performance (Graph 3). Only 1.2% indicated experiencing symptoms of schizophrenia, and 2.9% expressed symptoms of bipolar affective disorder. Out of the 172 participants, 22.1% of student nurses indicated experiencing feelings of self‐harm, with 8.7% choosing not to disclose. Additionally, 19.4% of participants disclosed experiencing feelings or thoughts of suicide or having engaged in self‐harm. Similar to emotional distress, symptoms of anxiety and stress were more prevalent in the younger age bracket of 18–29 years (84.7%), with the other two age categories, 30–39 and above 40 years, reporting similar prevalence of 68.4% and 60.9%, respectively.

### 3.3 Substance Use

In response to the question regarding their experiences with mental health or addiction issues, 43.9% of participants acknowledged having challenges. Over the past year, 8.1% reported struggling with substance use. Some students reported excessive alcohol use, cannabis use, nicotine use, vaping cannabis and some refusal to reveal substance use. Some of the free‐text responses in the survey relating to shared type of use and effects include:

‘Marijuana‐ relaxing and helps with anxiety. Alcohol‐ helps to unwind not good for overall health’.

‘Alcohol binge drinking which has caused me physical injury and ill health’.

‘Vaping, on special occasion recreational marijuana use. Getting to the end of every day thinking I need a wine or shot to refocus. It always calms me down, sometimes it feels so loud in me head its overwhelming. Then I feel guilty afterwards’.

‘Use of prescription and recreational drugs’.

‘Don't want to add’.

Although some context was provided in the responses, it was unclear in several comments on not wishing to disclose the specific substance use. It is possible this could be the result of fear, stigma and shame.

### 3.4 Physical Health

Upon rating their overall physical health, 61% of respondents indicated it to be good to excellent, while less than half (39%) considered it somewhat good to poor.

### 3.5 Mental Health Supports

In the section investigating mental health supports, 41.9% of respondents rated strategies such as physical exercise, socialising, mindfulness and art as beneficial for their mental well‐being. A comparable percentage (40.1%) of students found support from family/whānau or friends to be helpful. Other support factors included resilience strategies (9.3%); professional support from counsellors, psychologists or psychiatrists (4.7%); mental health services (1.7%); general practitioners (1.2%); as well as tertiary support services and nursing lecturers (1.1%) respectively. Pharmacological support was also reported, with 15.7% of participants indicating the use of medication to aid their mental health.

### 3.6 Qualitative Results

#### 3.6.1 Theme 1: Emotional Distress and Associated Effects

Emotional distress was an overarching theme. Participants cited a combination of both institutional and external factors impacting their mental health over the duration of their nursing programme. One student pointed out the increased emotional distress of being an international student in New Zealand. The challenges of adjusting to a foreign country, cultural differences and adapting to a new educational system can result in feelings of stress, depression and isolation. The student reported that,

Because I am an international student, there are many things that worry me. I have no family around, language barriers, and financial problems. I often feel lonely, and it makes me depressed and stressed when I have no one to talk to. (P40)

Emotional distress among nursing students was categorised into three prominent areas: anxiety, stress and depression. A significant number of participants disclosed struggling with all three of these phenomena over the past year. Some attributed their emotional struggles to pre‐existing conditions of anxiety and depression, while others pointed to the demanding workload of the nursing programme as a catalyst for these challenges as highlighted by two nurses' responses,

[I often feel] Sad, anxious, depressed, isolated, unsupported, tired out. I could continue. (P1)

Sad and anxious, anxiety, sad and stressed. Often with balancing academic/personal/work life, I can only give so much to each part of my life before I become burnt out. (P27)

##### 3.6.1.1 Anxiety and Stress

Student nurse participants reported experiencing symptoms of anxiety including an inability to relax, panic sensations and overall feelings of anxiousness. For some students, pre‐existing anxiety posed a significant mental health challenge, exacerbated by the demands of academic studies. The escalation of anxiety symptoms notably affected their ability to concentrate and absorb information, impacting their learning experience in both theoretical classes and clinical environments. Participants also highlighted the stress induced by academic assignments and deadlines, coupled with the fear of failure heightening their feelings of anxiety. Students specifically mentioned the disruptions and changes to the degree programme as sources of stress, ultimately affecting their overall well‐being. A few participants expressed significant shifts in their well‐being, describing feelings of disorientation, isolation and even instances of panic attacks. A few students explained,

I've always had anxiety and find that sometimes it can prevent me from attending class or being able to focus when my anxiety is bad. (P132)

Ever since I failed my essay at BN2, I always get anxious when I write future essays. It makes me anxious and I tend to panic if it's not good enough for a good mark. (P100)

Nearly a constant state of anxiety due to the ongoing disruptions and changes to our degree. There has been zero continuity in my education, and it has greatly impacted my well‐being. (P48)

Had a panic attack at work and ended up at ED (emergency department) with symptoms presenting as a heart attack. It was just a panic attack. (P123)

Some student nurses highlighted financial stress as a significant contributor to their reported anxiety symptoms. They faced the challenge of balancing the financial demands of living expenses while juggling the responsibilities of being a full‐time student and managing the workload of their courses. These dual pressures exacerbated feelings of anxiety, with one student commenting, ‘Balancing work and school‐ trying to earn enough money during placements is extremely tiring. Causing a lot of extreme stress and anxiety’ (P146). Another student reported

I can't financially support myself, I don't have food, I don't sleep, I can't focus. Some weeks I can't afford groceries, or petrol. Or money to pay for parking. It overwhelms me, I feel flooded with emotion. (P47)

##### 3.6.1.2 Depression

Many of the respondents discussed experiencing anxiety and depression together. However, some of the student nurses solely discussed depression as a factor impacting their emotional well‐being. Three participants described external contributors to their feelings of depression, such as relationship breakdowns and personal issues. P88 wrote, ‘Personal issues that have caused depression and a lack of focus’.

Depressive symptoms were diversely described and included an inability to concentrate, diminished motivation, fatigue, memory issues and a sustained sense of sadness. One student commented.

[My depression results in an] Inability to focus, lack of motivation, feeling sad frequently. (P114)

##### 3.6.1.3 Bipolar Affective Disorder

Five student nurses reported experiencing symptoms consistent with bipolar affective disorder (BPAD). Among them, two participants expressed challenges in managing their mental health symptoms, particularly those associated with BPAD, while navigating their academic studies.

My bipolar has become more difficult to manage. (P77)

Difficulty concentrating, feeling sad, lacking energy, guilt, self‐doubt. (P88)

Another student nurse shared their experiences of struggling with conflicting diagnoses, which caused uncertainty and stress as they attempted to adjust to changing diagnostic information. ‘[I have been] legally diagnosed with Bipolar type 2 but am showing more symptoms of bipolar 1 now’ (P11). This process of navigating diagnostic changes alongside ongoing study requirements presented additional challenges for them. Two student nurses related how symptoms of BPAD had significantly affected their daily lives and psychological well‐being. One student commented, ‘Hot and cold within milliseconds, I don't get too bad but sometimes I'm uncontrollably down in the dumps for weeks on end and struggle to get myself to do anything at all’ (P107).

##### 3.6.1.4 Schizophrenia/Psychosis

Two student nurses disclosed they experienced symptoms consistent with schizophrenia, which impacted their lives and academic journeys. ‘[I experience] auditory hallucinations’ (P18). Additionally, the two student nurses shared their experience of psychosis, characterised by active symptoms of both auditory hallucinations, procrastination and inability to focus:

My psychosis symptoms had made me procrastinate, unable to focus for more than one. Hour, unable to complete my daily tasks. That led me to feel depressed and anxious as I feel I'm not as good as the other students, but I know I'm different and I have psychosis, so I learn different, and I have to study differently’ (if it makes sense). (P12)

##### 3.6.1.5 Deliberate Self‐Harm and Suicidal Ideation

Some participants discussed self‐harm and suicidal ideation, with one student expressing fleeting thoughts of suicide,

Regarding having suicidal thoughts, that is as far as I go, sometimes just thinking about it make me feel guilty ‐ but I never consider self‐harming myself. Just the thought and feelings of disappearing and having that peace of mind, being understood, expressing feelings and being accepted is important for me ‐ by my family. Right now, just thinking about it… Is honestly making me feel emotional and feeling like I could suddenly burst into tears. (P12)

#### 3.6.2 Theme 2: Emotional and Psychological Impacts on Nursing Students' Academic Journey

Emotional and psychological factors significantly affected student nurses' ability to fully engage in their studies. Many participants reported that these challenges posed significant difficulties across various aspects of their degree studies. The primary issues raised by students as psychologically problematic centred on difficulties with concentration and motivation, which adversely affected their nursing studies.

##### 3.6.2.1 Concentration

Some student nurses highlighted the impact of psychological factors, particularly anxiety, on their ability to concentrate on coursework. They emphasised that underlying psychological conditions, including attention deficit hyperactivity disorder (ADHD), obsessional‐compulsive disorder (OCD), depression and anxiety significantly hampered their focus. Consequently, they resorted to less effective study practices such as cramming or disengagement; one participant described,

With (diagnosed) ADHD (attention deficit hyperactivity disorder) I struggle with emotional regulation and staying on track and will either be hyper focused or have no focus at all so it's very irregular and learning online is hard to stay on task. Recently I was diagnosed with OCD which has made daily life hard and due to getting very little sleep it affects my studies. (P9)

Furthermore, some participants reflected on how anxiety had a physiological impact, disrupting their sleep patterns and consequently affecting their ability to concentrate. One nurse discussed,

Some days, I'm not able to concentrate because of overwhelming emotions. My anxiety also affects my sleep to where I usually will only sleep for 5 hours and I'm always tired, so it is hard to learn. (P6)

Some students noted that external factors, such as financial stresses, social and personal relationships, as well as living arrangements, also influenced their concentration levels. One participant commented,

I understand nursing is a demanding course. However, compared to other courses, it is a lot more challenging to make time for social/family time as well as working part‐time. I am not able to work while I'm on placement; therefore, as well as focusing every day, which leaves me emotionally exhausted, I also have financial stress on how I'm going to make ends meet whilst my time on placement. This year, I experienced having to move three different times as well as trying to complete two large essays and group assignments that were all happening around the same time. (P135)

##### 3.6.2.2 Motivation

Several student nurse narratives highlighted the influence of their own mental health on their motivation levels toward studying. Some students expressed feeling overwhelmed by their thoughts and emotions, which hindered their ability to engage in academic studies or other aspects of their lives. This challenge in participating in daily activities persisted until they attained a level of mental well‐being essential for effective functioning; a process that often‐demanded time, which was not always readily available, especially with looming deadlines:

It makes me feel as though I am unable to do something. I feel like I am not good enough. It makes me rethink my life choices constantly even if I know I want to do something. When I feel like this, I often am not motivated to study, do work, see people or do anything productive with my life. (P28)

I tend to become overwhelmed with my thoughts and I shut down and just want to do nothing. It can take me a while to talk myself out of it or I can stay in that state for a day or two until I realise that I need to get my act together to complete the assignments. They usually do not turn out well. (P95)

A student singled out the pressures linked to workload management, highlighting how these pressures could negatively impact their mental well‐being, dampen motivation and potentially create financial strain:

Due to the pressure of school, in general it has resulted in me not wanting to work during the semester mainly because I felt as though I was not mentally ready. I would always make sure that I was mentally okay before going back to work. But honestly sometimes the course work can be overwhelming and in turn, it can cause you to isolate yourself. (P2)

#### 3.6.3 Theme 3 Tertiary Support Systems

##### 3.6.3.1 Student Awareness of Tertiary Mental Health Support Systems

Responses from students regarding their awareness of existing mental health support services varied. The primary services mentioned included counsellors, mainstream student support, cultural support, chaplaincy and lecturer support.

I found that student support and lecturers are also very helpful, (I am aware of the) MIT counselling service.(P169)

However, a portion of students reported a lack of awareness or negative attitudes regarding mental health support services within the institute.

I have tried to get support, but I found it very difficult to actually get what I needed. Whenever I try to book an appointment, they are always fully booked. (P21)

Yes, but they are incredibly poor. They are understaffed and under‐resourced to adequately take care of students. (P49)

##### 3.6.3.2 Future Supports for Student Nurses' Mental Health (What Helps?)

Student nurses discussed access to funded resources such as counselling and psychiatrist services as supportive measures when experiencing emotional distress or mental health challenges. However, student nurses also expressed the financial burden associated with accessing these psychological services, making it challenge them to get help due to financial constraints.

I think the available services need to be more encouraged, especially for nurses because of the workload. Hopefully in future, when we do become RNs, we will know how to deal with our mental health in the nursing field. (P3)

Another key suggestion from students was the need for more skilled and supportive lecturers, along with an increased number of lecturers with an awareness to addressing students' mental health needs throughout their academic journey. P44 wrote, ‘[we need] more support from lecturers. I didn't even know there was a counsellor so more education and support’.

Implementing mental health workshops, establishing chat rooms, LGBTQ support and displaying mental health posters and cards across the campuses were suggested. Additionally, providing focused mental health resilience training throughout all BN years and offering physical and social activities to support mental health were recommended. Increasing mental health awareness and teaching from earlier in the course was also proposed.

There could be a survey sent out either before [beginning] the course, midway through to see how they are doing [mental well‐being]. I know it's a lot of work but I feel this might help some students that are afraid or too shy to reach out. (P128)

## 4 Discussion and Recommendations

The study's primary aim was to explore the rates and associated experiences among student nurses regarding mental health and substance use challenges within a tertiary institution. It also aimed to identify available support services and barriers to accessing those services. The findings of the present study highlight a substantial impact of emotional distress and poor mental health on student nurses, with anxiety and stress emerging as predominant challenges, particularly among younger students, potentially jeopardising their mental well‐being and academic successes. Previous research also echoed the notably higher levels of psychological distress in tertiary students compared to the general population, underlining the necessity for early interventions to mitigate the progression to more severe mental illness (Stallman 2010). Similarly, Fitzpatrick, Harris, and Drawve (2020) discussed the vulnerability of specific groups including females, ethnic minorities and young adults with prior mental health challenges, who face an elevated risk of suicide.

A substantial proportion of participants in the current study reported instances of self‐harm, suicidal ideation, and substance use, mirroring findings from previous research that linked these behaviours along with ADHD, and personality disorders with academic performance indicators (Kitzrow 2003). A study in Brazil revealed that 12.3% of nursing students experienced suicidal ideation, suggesting challenges in adapting to academic demands (Moraes et al. 2021). Similarly, the current study highlighted student nurses' struggles with overwhelming study schedules and academic expectations, impacting their mental well‐being.

Recent data from New Zealand's chief coroner for the year up to June 2023 revealed a suspected suicide rate of 10.6 per 100 000 people, a slight increase from the previous year's rate of 10.5 per 100 000 (Ministry of Health NZ 2024). Within the context of this study, instances of suicidal ideation and self‐harm among student nurses during their academic journey emphasise the significant psychological distress experienced in this cohort. This finding highlights the vulnerability of student nurses as they navigate the balance between their responsibilities in caring for the community, managing their studies and coping with serious mental health challenges. It is imperative that both the relevant statistics and voices of students are urgently recognised and heard by educational institutions. Failure to provide appropriate support may severely impact students' ability to cope with feelings of despair, potentially leading to self‐injury or suicide.

In the present study, 8.1% of student nurses reported using substances to cope with study‐related difficulties and anxieties. As already described marijuana, alcohol, vaping and non‐disclosed (possible illicit substances). Previous research suggests rates of addiction among nurses can be as high as 20% (Monroe and Kenaga 2011). Boulton and O'Connell (2017) noted that substance use often begins during or before nursing education. They found higher stress levels among student nurses correlated with increased substance use, while those perceiving strong faculty support tended to use fewer stimulants. Although the current study reported lower substance use rates, factors like stigma and professional apprehension may have led to underreporting, as acknowledged in earlier studies (Boulton and O'Connell 2017; Monroe and Kenaga 2011).

The survey identified that 1.2% (*n* = 2) of student nurses experienced psychosis symptoms, including auditory hallucinations, and some associative physical symptoms, such as headaches, impacting their lives and study. Additionally, 2.9% (*n* = 5) reported symptoms or a clinical diagnosis of BPAD, facing challenges in symptom management while studying. These findings align with previous research by Maassen et al. (2018), indicating challenges in social recovery, cognitive functioning and stigma concerns, as well as the reluctance observed in the current study regarding the disclosure of substance use.

Ryan and Deci (2000) emphasised motivation as the driving force behind task initiation, providing direction and purpose in achieving educational goals. The current study highlighted the adverse impact of poor mental health on nursing students' motivation and concentration, hindering their academic engagement and daily activities. Furthermore, it identified barriers to accessing institutional support services, leading many students to rely on self‐management strategies or informal networks for assistance. In the demanding environment of health care education, student nurses often face immense pressure to excel academically, posing challenges to their motivation.

The study emphasises the critical need for robust mental health support within education institutions to prioritise student well‐being. Addressing the unique challenges encountered by nursing students is essential for improving their academic success and overall welfare. Tailored interventions and support services play a vital role in navigating the intricate interplay between mental health and academic achievement. While some students in the current study acknowledged the presence of mental health support services within their institution, others found them to be insufficient or ineffective. This aligns with previous research, which discusses disparities in mental health services across New Zealand tertiary providers, suggesting that some institutions lack the resources or commitment to adequately support student mental health (Happell et al. 2019; Limpus and Carlyon 2019). Additionally, some students in the current study reported a lack of awareness of institutional support services, mirroring findings from Nazari and van Ommen (2019), who also identified stigma and confidentiality concerns as barriers to accessing support in New Zealand institutions. Overcoming these barriers requires concerted efforts to raise awareness, improve accessibility, and challenge stigmatising attitudes. By prioritising mental health support and fostering a supportive environment, educational institutions can better serve their student population.

### 4.1 Implications for Practice

This study signals a pressing need for proactive measures to promote improved mental health and well‐being among undergraduate nursing students. The identified areas of concern emphasise the urgency for action and implementation of changes required within the nursing education curriculum. The recommendations outlined in Table 2, delineate key strategies aimed at enhancing the mental health of undergraduate nursing students.

**TABLE 2:** Strategies for enhancing mental health.

Strategy	Rationale
Mental illness prevention	Early intervention and mental strengths training should be integrated throughout the BN programme, spanning from the first to the final year. This approach entails resilience‐building exercises and fostering the ability to identify mental health symptoms
Recovery focus	Emphasising a recovery‐orientated approach to mental health care within the curriculum
Integrated services	Efforts should be made to expand access to mental health services including therapy, counselling, primary mental health services, general practitioner collaboration and e‐therapy
Reducing stigma	Addressing mental health stigma from the outset of academic nursing studies is essential. Health literacy initiatives, such as mental health first aid training, can help dispel misconceptions and foster positive attitudes toward mental health
Lecturer support and training	Academic lecturers should undergo training to raise awareness of mental health issues and ensure students have accurate information and access to support services
Education	Integrating ‘Expert By Experience’ (EBE) training in mental health nursing education. EBE is necessary to help student nurses comprehend person‐centred care for service users. Students' experience and communication skills improve; and stigma, prejudice and preconceptions about mental health issues decrease (Happell et al. 2022)

The study highlights the importance of collaboration between tertiary institutions and universities to support the mental health of nursing students. Integrating psych‐educational sessions into the curriculum, including mental strength‐building exercises, cognitive behavioural techniques, mindfulness practices and mental health first aid training, can reduce stigma and enhance health literacy. This approach fosters positive attitudes and equips students with coping strategies, ultimately improving their well‐being and academic success.

### 4.2 Limitations/Contextual Considerations

The findings need to be considered in the light of limitations. It is important to note that the questionnaire was developed for a one‐time application at a specific school of nursing in the Auckland region, involving a relatively small cohort. Therefore, caution should be exercised when considering the generalisability of the findings to the broader undergraduate nursing student population. Although the results may suggest a trend, they are not broadly applicable beyond the studied group. In addition, the focus of the survey is on a New Zealand context and may not represent other demographics or cultures internationally.

Students were under pressure with their studies as shown; therefore, had limited time to engage in research. Even if they chose to engage, they may have been physically and emotionally exhausted which may be why not all questions were answered or why some responses were limited. Only two participants, however, omitted single answers in two categories.

It must also be acknowledged that participants' experiences and understanding of mental health might not fully mirror those of all student nurses per se. Caution is needed with regard to generalisability of the study's findings.

## 5 Conclusion

Given the growing New Zealand population and the subsequent surge in demand for nursing professionals, educational institutions must prioritise the mental well‐being of student nurses. The study's key findings highlighted that a significant proportion of nursing students in the institute experienced mental health challenges encompassing emotional distress, anxiety, depression, other major mental health disorders and substance use. There is a need for proactive measures, including early intervention and the seamless integration of comprehensive mental health training across the nursing curriculum. Furthermore, there is a compelling need for enhanced education and support for academic faculty to effectively address the multifaceted mental health needs of student nurses. Of primary concern is the apparent inadequacy and underutilisation of current support services tailored for student nurses. Persistent emotional distress hinders students' educational pathways and poses potential implications for their future roles as registered nurses. Equipping them with adequate skills and support to navigate mental health challenges is essential for their success during their education and beyond.

## 6 Relevance to Clinical Practice

This study emphasises the importance of taking proactive measures to enhance the mental health and well‐being of undergraduate nursing students. The highlighted areas of concern indicate the necessity for swift action and implementation of changes required within the nursing education curriculum. Addressing the mental health and addiction challenges experienced by nursing students is imperative, given their potential adverse effects on academic success and overall well‐being. These efforts will cultivate stronger, more resilient nurses, ultimately benefitting both practice and the well‐being of our future workforce.

## Author Contributions

Percentage of work each researcher has contributed to the study.

## Conflicts of Interest

The authors declare no conflicts of interest.

## Data Availability

The authors and the research team have full access to all the data in the study and takes responsibility for the integrity of the data and the accuracy of the data analysis.

## References

[inm13464-bib-0002] Al‐Ababneh, M. 2020. “Linking Ontology, Epistemology and Research Methodology.” Science & Philosophy 8: 75–91.

[inm13464-bib-0006] Boulton, M. , and K. A. O'Connell . 2017. “Nursing Students' Perceived Faculty Support, Stress, and Substance Misuse.” Journal of Nursing Education 56: 404–411. 10.3928/01484834-20170619-04. 28662256

[inm13464-bib-0008] Brown, J. S. L. 2018. “Student Mental Health: Some Answers and More Questions.” Journal of Mental Health 27: 193–196. 10.1080/09638237.2018.1470319. 29768071

[inm13464-bib-0009] Campbell, F. , L. Blank , A. Cantrell , et al. 2022. “Factors That Influence the Mental Health of University and College Students in the UK: A Systematic Review.” BMC Public Health 22: 1778. 10.1186/s12889-022-13943-x. 36123714 PMC9484851

[inm13464-bib-0011] Choi, H. , B. Hwang , S. Kim , H. Ko , S. Kim , and C. Kim . 2016. “Clinical Education in Psychiatric Mental Health Nursing: Overcoming Current Challenges.” Nurse Education Today 39: 109–115. 10.1016/j.nedt.2016.01.021. 27006041

[inm13464-bib-0012] Cooper, D. R. , and P. S. Schindler . 2014. Business Research Methods. 12th ed. New York: McGraw Hill.

[inm13464-bib-0013] Corrigan, P. W. , B. G. Druss , and D. A. Perlick . 2014. “The Impact of Mental Illness Stigma on Seeking and Participating in Mental Health Care.” Psychological Science in the Public Interest 15: 37–70. 10.1177/1529100614531398. 26171956

[inm13464-bib-0014] Corrigan, P. W. , and D. Rao . 2012. “On the Self‐Stigma of Mental Illness: Stages, Disclosure, and Strategies for Change.” Canadian Journal of Psychiatry / Revue Canadienne de Psychiatrie 57: 464–469. 10.1177/070674371205700804. 22854028 PMC3610943

[inm13464-bib-0018] Fitzpatrick, K. M. , C. Harris , and G. Drawve . 2020. “How Bad Is It? Suicidality in the Middle of the COVID‐19 Pandemic.” Suicide & Life Threatening Behaviour 50: 1241–1249. 10.1111/sltb.12655. PMC7361329 32589799

[inm13464-bib-0019] Foster, K. , J.‐A. Giandinoto , T. Furness , A. Blanco , E. Withers , and L. Alexander . 2021. “‘Anyone Can Have a Mental illness’: A Qualitative Inquiry of Pre‐Registration Nursing Students' Experiences of Traditional Mental Health Clinical Placements.” International Journal of Mental Health Nursing 30: 83–92. 10.1111/inm.12808. 33145951

[inm13464-bib-0020] Foster, K. , E. Withers , T. Blanco , et al. 2019. “Undergraduate Nursing Students' Stigma and Recovery Attitudes During Mental Health Clinical Placement: A Pre/Post‐Test Survey Study.” International Journal of Mental Health Nursing 28: 1068–1080. 10.1111/inm.12634. 31338978

[inm13464-bib-0021] Gaber, J. 2010. “Face Validity.” In Encyclopedia of Research Design, 471–474. Thousand Oaks, CA: SAGE Publications. 10.4135/9781412961288.

[inm13464-bib-0022] Happell, B. , S. Waks , J. Bocking , et al. 2019. “I Felt Some Prejudice in the Back of My Head: Nursing Students' Perspectives on Learning About Mental Health From Experts by Experience.” Journal of Psychiatric and Mental Health Nursing 26: 233–243. 10.1111/jpm.12540. 31220380

[inm13464-bib-0023] Happell, B. , T. Warner , S. Waks , et al. 2022. “Something Special, Something Unique: Perspectives of Experts by Experience in Mental Health Nursing Education on Their Contribution.” Journal of Psychiatric and Mental Health Nursing 29: 346–358. 10.1111/jpm.12773. 34032356

[inm13464-bib-0024] Hemingway, S. , A. Clifton , and K.‐L. Edward . 2016. “The Future of Mental Health Nursing Education in the United Kingdom: Reflections on the Australian and New Zealand Experience.” Journal of Psychiatric and Mental Health Nursing 23: 331–337. 10.1111/jpm.12312. 27307264

[inm13464-bib-0025] Hudson, M. , M. Milne , P. Reynolds , K. Russell , and B. Smith . 2018. “Te Ara Tika Guidelines for Māori Research Ethics: A Framework for Researchers and Ethics Committee Members.” https://www.hrc.govt.nz/sites/default/files/2019‐06/Resource%20Library%20PDF%20‐%20Te%20Ara%20Tika%20Guidelines%20for%20Maori%20Research%20Ethics_0.pdf.

[inm13464-bib-0028] Kessler, R. C. , G. P. Amminger , S. Aguilar‐Gaxiola , J. Alonso , S. Lee , and T. B. Ustün . 2007. “Age of Onset of Mental Disorders: A Review of Recent Literature.” Current Opinion in Psychiatry 20: 359–364. 10.1097/YCO.0b013e32816ebc8c. 17551351 PMC1925038

[inm13464-bib-0029] Kitzrow, M. A. 2003. “The Mental Health Needs of Today's College Students: Challenges and Recommendations.” Journal of Student Affairs Research and Practice 41: 165–179.

[inm13464-bib-0030] Ladejo, J. 2021. “A Thematic Analysis of the Reported Effect Anxiety Has on University Students.” Education and Urban Society 55: 289–313. 10.1177/00131245211062512.

[inm13464-bib-0031] Limpus, W. , and T. Carlyon . 2019. “Considering How Tertiary Education Providers Can Best Support the Mental Health and Wellbeing of Their Students.” JANZSSA: Journal of the Australian and New Zealand Student Services Association 27: 188–200. 10.3316/informit.378964648666713.

[inm13464-bib-0032] Lipson, S. K. , and D. Eisenberg . 2017. “Mental Health and Academic Attitudes and Expectations in University Populations: Results From the Healthy Minds Study.” Journal of Mental Health 27: 205–213. 10.1080/09638237.2017.1417567. 29265935

[inm13464-bib-0033] Maassen, E. F. , B. J. Regeer , E. J. Reeger , J. F. G. Bunders , and R. W. Kupka . 2018. “The Challenges of Living With Bipolar Disorder: A Qualitative Study of the Implications for Health Care and Research.” International Journal of Bipolar Disorder 6: 23. 10.1186/s40345-018-0131-y. PMC6218397 30397833

[inm13464-bib-0034] McIntyre, J. C. , J. Worsley , R. Corcoran , P. H. Woods , and R. P. Bentall . 2018. “Academic and Non‐academic Predictors of Student Psychological Distress: The Role of Social Identity and Loneliness.” Journal of Mental Health 27: 230–239. 10.1080/09638237.2018.1437608. 29436883

[inm13464-bib-0035] Ministry of Health NZ . 2024. “Understanding Suicide Data.” https://www.health.govt.nz/our‐work/mental‐health‐and‐addiction/suicide‐prevention‐new‐zealand/understanding‐suicide‐data.

[inm13464-bib-0038] Monroe, T. , and H. Kenaga . 2011. “Don't Ask Don't Tell: Substance Abuse and Addiction Among Nurses.” Journal of Clinical Nursing 20: 504–509. 10.1111/j.1365-2702.2010.03518.x. 21040041 PMC6415967

[inm13464-bib-0039] Moraes, S. M. A. B. , V. F. B. Barbosa , A. C. S. Alexandre , S. C. D. Santos , F. J. Guimarães , and J. L. A. Veras . 2021. “Risk of Suicide Among Nursing Students.” Revista Brasileira de Enfermagem 74: 6. 10.1590/0034-7167-2020-0867. 34431934

[inm13464-bib-0040] Najafi, F. , F. K. Saravi , A. Navidian , and S. M. Raeisi . 2018. “The Relationship Between Internet Addiction, Loneliness and Sleep Quality Among Students of Nursing and Midwifery Faculty.” Zahedan Journal Research Medical Science 20: e68394. 10.5812/zjrms.68394.

[inm13464-bib-0041] Nazari, V. , and C. van Ommen . 2019. “‘It's an Important Thing and Can Change Someone Without You Realising’: New Zealand Students' Views of University Mental Health Services.” New Zealand Journal of Psychology 48, no. 2: 52–63.

[inm13464-bib-0046] Ryan, R. M. , and E. L. Deci . 2000. “Intrinsic and Extrinsic Motivations: Classic Definitions and New Directions.” Contemporary Educational Psychology 25: 54–67. 10.1006/ceps.1999.1020. 10620381

[inm13464-bib-0051] Stallman, H. 2010. “Psychological Distress in University Students: A Comparison With General Population Data.” Australian Psychologist 45, no. 4: 249–257.

[inm13464-bib-0053] Teddlie, C. , and A. Tashakkori . 2009. Foundations of Mixed Methods Research: Integrating Quantitative and Qualitative Approaches in the Social and Behavioural Sciences. Thousand Oaks, CA: Sage.

[inm13464-bib-0054] Thomas, D. R. 2006. “A General Inductive Approach for Analyzing Qualitative Evaluation Data.” American Journal of Evaluation 27: 237–246. 10.1177/1098214005283748.

